# Transgenerational Variations in DNA Methylation Induced by Drought Stress in Two Rice Varieties with Distinguished Difference to Drought Resistance

**DOI:** 10.1371/journal.pone.0080253

**Published:** 2013-11-11

**Authors:** Xiaoguo Zheng, Liang Chen, Mingshou Li, Qiaojun Lou, Hui Xia, Pei Wang, Tiemei Li, Hongyan Liu, Lijun Luo

**Affiliations:** 1 College of Plant Sciences & Technology, Huazhong Agricultural University, Wuhan, China; 2 Shanghai Agrobiological Gene Center, Shanghai, China; Duke University, United States of America

## Abstract

Adverse environmental conditions have large impacts on plant growth and crop production. One of the crucial mechanisms that plants use in variable and stressful natural environments is gene expression modulation through epigenetic modification. In this study, two rice varieties with different drought resistance levels were cultivated under drought stress from tilling stage to seed filling stage for six successive generations. The variations in DNA methylation of the original generation (G0) and the sixth generation (G6) of these two varieties in normal condition (CK) and under drought stress (DT) at seedling stage were assessed by using Methylation Sensitive Amplification Polymorphism (MSAP) method. The results revealed that drought stress had a cumulative effect on the DNA methylation pattern of both varieties, but these two varieties had different responses to drought stress in DNA methylation. The DNA methylation levels of II-32B (sensitive) and Huhan-3 (resistant) were around 39% and 32%, respectively. Genome-wide DNA methylation variations among generations or treatments accounted for around 13.1% of total MSAP loci in II-32B, but was only approximately 1.3% in Huhan-3. In II-32B, 27.6% of total differentially methylated loci (DML) were directly induced by drought stress and 3.2% of total DML stably transmitted their changed DNA methylation status to the next generation. In Huhan-3, the numbers were 48.8% and 29.8%, respectively. Therefore, entrainment had greater effect on Huhan-3 than on II-32B. Sequence analysis revealed that the DML were widely distributed on all 12 rice chromosomes and that it mainly occurred on the gene’s promoter and exon region. Some genes with DML respond to environmental stresses. The inheritance of epigenetic variations induced by drought stress may provide a new way to develop drought resistant rice varieties.

## Introduction

Recent studies revealed that epigenetic modifications, such as DNA methylation and histone modification, play an important role in plant development and in resistance to environmental stresses [1,2]. DNA methylation is a conserved epigenetic marker that mainly occurs at the cytosine bases in all plants’ sequence contexts: symmetric CG and CHG contexts (in which H = A, T or C) and in an asymmetric CHH context [3,4]. It is associated with many important biological processes, including heterochromatin formation, defense against transposon proliferation, genomic imprinting, regulation of endogenous gene expression, and silencing of transgenes [5-9]. CpG dinucleotides are usually clustered around the regulatory region of genes, especially in the promoters and first exons, which can affect their transcriptional regulations [10]. 

Epigenetic variations in natural plant populations help individuals to cope with different environments and some of the variations are heritable [11]. It is reported that variations in DNA methylation can be inherited across at least eight generations in the absence of extensive DNA sequence polymorphisms and without selection in Arabidopsis [12]. Specific stress can induce particular DNA methylation changes in asexual dandelions and most of the induced changes are faithfully transmitted to offspring [13]. Biotic and abiotic stress, such as bacterial pathogens, avirulent bacteria, salicylic acid, salinity and drought can induce widespread dynamic DNA methylation in plants [14-20]. Akimoto et al. (2007) reported that rice plant treated by 5-azadeoxycytidine acquired disease resistance by abolishing the constitutive silencing of Xa21G through promoter demethylation and that disease resistance could be stably inherited [21]. In addition, histone H3 threonine 3 (H3T3) phosphorylation was necessary for heritable epigenetic silencing in Chlamydomonas [22]. Under dehydration stress conditions, H3K4me3 levels were shown to be positively correlated with the change in transcript levels, while H3K4me1 levels were negatively correlated with the change in transcript levels in Arabidopsis [23]. Thus, DNA methylation and other epigenetic variations, such as histone modifications, add another layer of heritable epigenetic changes and provide a source of heritable phenotypic variation that is not caused by variance in the DNA sequence [5,12,24-29].

Rice is one of the most important model species for cereals and other monocotyledonous plants due to its small genome size. It is also one of the world’s most important staple foods, providing 30% of the calories consumed in Asian countries [30]. However, its yield is limited by several abiotic and biotic stresses, such as drought, diseases, pests, salinity and more. Drought stress is a major environmental factor that reduces rice production by 13–35% in most rain-fed systems worldwide [31,32]. Drought resistance is a complex agronomic trait. Rice has three different strategies to minimize drought stress inﬂuence: dehydration avoidance, the plant’s capacity to sustain high water status in dry conditions; dehydration tolerance, the relative capacity of plants to maintain function under low leaf water status; and drought recovery, the recovery capability of plants after a period of severe drought [33]. When studying dehydration tolerance in plants, polyethylene glycol (PEG) is usually used as a permeable mass to simulate water stress (osmotic stress). PEG mitigates the mechanism of plants’ dehydration avoidance and other natural environmental factors [34-37]. Rice varieties differ greatly in their resistance to drought. Conventional genetic research methods about drought resistance and other agronomic traits are mainly based on genomic variations, such as Quantities Trait Loci (QTL) [38-41]. Other methods, such as microarrays, examine variation in the gene expression level [42]. Some drought resistance related genes have been cloned [43-45]. However, studies involving variations in the epigenetic modification of rice induced by drought stress are scarce [14,46]. Additionally, the cumulative effect of multigenerational drought stress on DNA methylation in rice has never been investigated. 

In the present study, a drought-sensitive variety (II-32B) and a drought-resistant cultivar (Huhan-3) were grown under drought stress for six successive generations. We posed constant drought stress on rice as a selection force to imitate natural selection in severe environments. Because of the anthropogenic factors, this method might be better defined as drought entrainment rather than natural selection. Genome-wide variations in DNA methylation on CCGG sites were assessed for the original generation (G0) and the sixth generation (G6) under normal water condition (CK) and drought stress (DT) which was imitated by 20% PEG6000 treatment at the seedling stage using Methylation Sensitive Amplification Polymorphism (MSAP) method. This study asked: 1. What cumulative DNA methylation pattern changes exist after being cultivated under drought stress for six generations? (Cumulative effect?) 2. Does a random or directional trend exist in DNA methylation pattern variation induced by drought stress? (Directional variation?) 3. Are the changes in DNA methylation induced by drought stress preserved in subsequent generation of G6? (Transgenerational?) 4. Do variations in DNA methylation differ between two varieties with disproportionate drought resistance? 

## Materials and Methods

### Plant materials

Two rice varieties, II-32B (*Oryza sativa* L. ssp. *indica*) and Huhan-3 (*Oryza sativa* L. ssp. *japonica*), were used in this experiment. II-32B is a commonly used rice maintainer line in breeding and it is highly sensitive to drought stress, whereas Huhan-3 is a water-saving and drought-resistant rice (WDR) variety [33]. 

Seeds from a single individual plant of II-32B and Huhan-3 were designated as the starting generation G0. These were cultivated in the same experimental conditions, while drought stress was imposed on plants from the tilling stage to seed filling stage where drought stress has the largest impact on plant development and can cause severe yield loss. Seeds harvested from such drought stress treated plants were propagated for another five generations to obtain the G6 seeds. The G0 and G6 seeds of II-32B and Huhan-3 were used in this experiment. 

### Experimental treatments

This study used two experimental treatments: normal cultivation (CK) and cultivation under drought stress (DT). In the CK treatment, about 40 G0 and G6 seeds of II-32B and Huhan-3 were germinated at 23 °C in the incubator for 48 h, respectively, and their seedlings were cultivated for four weeks with sufficient nutrient solution in the environmental growth chamber (CONVIRON CMP6050). The temperature was set from 21°C to 29 °C, continuous light (200 mmol/m^2^/s) was set from 7 AM to 7 PM (12 h), and the humidity was set from 75% to 80%. In the DT treatment, the same amounts of seeds were cultivated for three weeks as in the CK treatment. These were then cultivated in the 20% solution of PEG 6000 for another week to imitate drought stress. 12 seedlings served as an experimental replicate and 3 replicates were used in this study. Leaf samples from eight seedlings of each replicate of both cultivars in two treatments were collected for MSAP analysis, respectively.

### DNA extraction and MSAP analysis

Genomic DNA was extracted from 24 mixed seedlings using the improved CTAB method modified from Murray and Thompson (1980) [47].

The MSAP approach is very similar to the standard AFLP [48]. Two methylation-sensitive restriction enzymes (*Msp*Ⅰ and *Hpa*Ⅱ) were used as frequent cutters and were combined with the same rare cutter (*EcoR*Ⅰ) in parallel batches, respectively. With some modifications to increase the number of amplified fragments and improve fingerprint readability, the MSAP was performed following the general steps described by Xiong et al. (1999) [49]. 

### Digestion reaction

The digestion and ligation reaction were separately performed. In the digestion reaction, DNA samples were separately digested with double enzyme combinations, *EcoR*Ⅰ/*Msp*Ⅰ and *EcoR*Ⅰ/*Hpa*Ⅱ. The reaction solution contains 250 ng genomic DNA, 2 μl 10 × T4 DNA ligase buffer (Promega), 10 U *EcoR*Ⅰ, 10 U *Msp*Ⅰ (or *Hpa*Ⅱ) (New England Biolabs, NEB), adding ddH_2_O to a final volume of 20 μl, subsequently incubated at 37°C for 2 h. 5 μl digestion product was checked with 0.5% agarose gels to confirm the DNA template was completely digested.

### Ligation reaction

Then, 15 μl digestion product was mixed with 5 pmol *EcoR* I adapter (5’- CTCGTAGACTGCGTACC-3’, 5’-AATTGGTACGCAGTCTAC-3’), 50 pmol *Hpa* II /*Msp* I (H/M) adapter (5’-GACGATGAGTCTAGAA-3’, 5’- CGTTCTAGACTCA- TC-3’), 1.5 U T4 ligase and 1.5 μl 10 x T4 ligation buffer. ddH_2_O is added to a final volume of 30 μl and incubated at 16 °C for 2h to overnight for ligation reaction. Enzymes were afterwards denatured at 65 °C for 10 min. Negative control samples were included at all steps to prevent contamination. The resultant products were diluted 20-fold and used as templates in the following pre-amplification.

### Pre-amplification

Pre-amplification was conducted in a 20 μl volume with 2 μl 10× PCR reaction buffer (Tiangen), 1 μl dNTPs (2.5 mM), 1.5 U Taq polymerase, 5 μl diluted product (as DNA template), and 5 μM pre-amplification primers (E1 5’-GACTGCGTACCAATTCA-3’, HM1 5’-GATGAGTCTA- GAACGGT-3’), adding ddH_2_O to 20 μl. The reaction was catalyzed for 29 cycles in a thermocycler of 94 °C 30 s, 56 °C 30 s, and 72 °C 1 min with a 72 °C 10 min final extension. 

### Selective amplification

The primers used in selective amplification are listed in Table 1. 20 μl volume for the selective amplification containing 2 μl 10× PCR reaction buffer, 1 μl dNTPs (2.5 mM), 1.5 U Taq polymerase, 1 μl pre-amplification product (as DNA template) and 1 μl *EcoR* I selective amplification primer (10 μM), 1 μl H/M selective amplification primer (10 μM) and 13.5 μl ddH_2_O. The selective amplification was performed with a touchdown program of 94 °C for 30 s, 65 °C for 30 s and 72 °C for 1 min, decreasing the annealing temperature by 0.7 °C per cycle during 12 cycles and then 24 cycles of 94 °C for 30 s, 56 °C for 30 s and 72 °C for 1 min with a final extension of 10 min at 72 °C. The final products were separated using 6% polyacrylamide gels and visualized via silver staining. 

**Table 1:** Selective primers used in MSAP analysis.

*Hpa* II/*Msp* I primer	Sequence (5’-3’)	*EcoR* I primer	Sequence (5’-3’)
HM31	GATGAGTCTAGAACGGTAA	E01	GACTGCGTACCAATTCATA
HM32	GATGAGTCTAGAACGGTAG	E02	GACTGCGTACCAATTCATG
HM33	GATGAGTCTAGAACGGTAC	E03	GACTGCGTACCAATTCATC
HM34	GATGAGTCTAGAACGGTAT	E04	GACTGCGTACCAATTCAGA
HM35	GATGAGTCTAGAACGGTGA	E05	GACTGCGTACCAATTCAGC
HM36	GATGAGTCTAGAACGGTGT	E06	GACTGCGTACCAATTCAGT
HM37	GATGAGTCTAGAACGGTGG	E07	GACTGCGTACCAATTCACA
HM38	GATGAGTCTAGAACGGTGC	E08	GACTGCGTACCAATTCACG
HM39	GATGAGTCTAGAACGGTCA	E09	GACTGCGTACCAATTCACT
HM310	GATGAGTCTAGAACGGTCT	E10	GACTGCGTACCAATTCACC
HM311	GATGAGTCTAGAACGGTCG	E11	GACTGCGTACCAATTCATT
HM312	GATGAGTCTAGAACGGTCC	E12	GACTGCGTACCAATTCAGG
HM313	GATGAGTCTAGAACGGTTA	E13	GACTGCGTACCAATTCAAG
HM314	GATGAGTCTAGAACGGTTG	E14	GACTGCGTACCAATTCAAC
HM315	GATGAGTCTAGAACGGTTC	E15	GACTGCGTACCAATTCAAT
HM316	GATGAGTCTAGAACGGTTT	E16	GACTGCGTACCAATTCAAA

Totally, 256 primer-pair combinations were used in the present study

### Band scoring and data analysis

The two isoschizomers (*Msp*Ⅰand *Hpa*Ⅱ) recognize the same sequence (5’-CCGG-3’) but differ in their sensitivity to DNA methylation [50].The scoring of differential methylation status on a specific site is based on the presence (scored as 1) or absence (scored as 0) of bands in the *EcoR*Ⅰ/*Msp*Ⅰ and *EcoR*Ⅰ/*Hpa*Ⅱ lanes. Comparing the two profiles of these two lanes allows for the assessment of the methylation status of the restriction sites. The full methylation sites (methylation at the internal C residue of both strands, MeCpG) is only recognized by *Msp* I with the band type represented by (1, 0). Meanwhile, plant-specific hemi-MeCpCpG sites (methylation at the external C residue in one DNA strand but not in its complement strand) is only recognized by *Hpa* II with the band type represented by (0, 1). Sites that are hypermethylated at both the internal and external Cs and sites that are fully methylated at the external Cs on both strands are cut by neither two enzymes and that band type is represented by (0, 0). The sites that are free from methylation are recognized by both isoschizomers with the band type represented by (1–1). In total, there are 4 types of band combinations in the two lanes that represent 4 types of DNA methylation statuses of the restriction sites (5’-CCGG-3’) (Table 2).

**Table 2:** Activity of restriction enzyme and the types of band combinations.

Type	I(1,1)	II(1,0)	III(0,1)	IV(0,0)	
	Free-methylation	Full-methylation	Hemi-methylation	Hype-methylation	
Sequence	CCGG	CCGG	CCGG	CCGG	CCGG
	GGCC	GGCC	GGCC	GGCC	GGCC
*Msp*Ⅰ	CC GG	CC GG	non	non	non
	GG CC	GG CC	non	non	non
HpaⅡ	CC GG	non	CC GG	non	non
	GG CC	non	GG CC	non	non

C: methylated cytosine.

The general DNA methylation level (%) was analyzed based on two strands of DNA and the following formula was calculated: (II*2+III*1+IV*2) / [(I+II+III+IV)*2]*100%. Any variations in DNA methylation between generations or treatments could be detected by comparing the methylation status of the corresponding samples. 

The proportions of the four band types were calculated using all samples. SPSS ver. 20.0 independent t-test was used to analyze differences between the two varieties’ band type proportions. 

### Cloning, sequencing and annotation of differentially methylated fragments

A set of 92 and 55 randomly selected fragments for II-32B and Huhan-3 were isolated, re-amplified with appropriate selective primer combinations, and purified, respectively; they showed differential methylation between generations or between treatments. Afterwards, the purified DNA fragments were cloned into DH5α using a commercial cloning kit (TransGene, Beijing, China) and sequenced at BGI. The sequences were used as query searches against the nucleotide databases of Grammene (http://www.gramene.org/) for homology and function annotation.

### mRNA quantiﬁcation by qPCR

Total RNA was extracted from three seedlings with TRNzol-A^+^ Total RNA Reagent (TIANGEN, Beijing, China). cDNA was culled from total RNA with PrimeScript^®^ RT reagent Kit (Takara Biotechnology, Dalian, China) according to the manufacturer’s instructions. Three experimental replicates and three technology replicates were included in our study. Oligoprimers are described in table S1. Real-time PCR was performed using Hard-Shell^®^ 96-Well PCR Plates (BIO-RAD, USA) with the CFX96^TM^ Real-Time System (BIO-RAD, USA). Each reaction contained 10 μl of 2 x SYBR Premix Ex Taq^TM^ (Takara Biotechnology, Dalian, China), 20 ng cDNA, and 0.1 μM gene-specific primers in a final volume of 20 μl. The thermal cycle used was 95°C for 30 s; then 40 cycles at 95°C for 5 s, and 60°C for 31 s, with an additional dissociation stage. The qPCR data were normalized to the expression of the housekeeping Actin gene in rice and after normalization, the data were presented as fold change relative to the 1 point.

## Results

### Two varieties had different amplified bands

**Figure 1:**
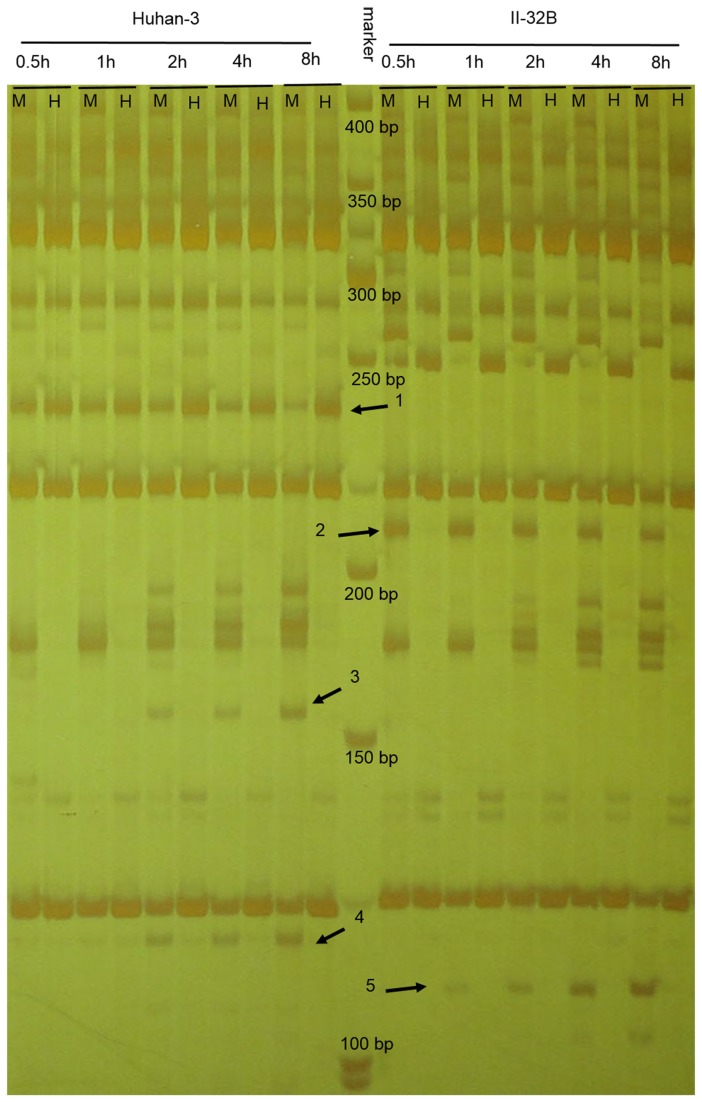
Selective amplification with primer-pair of E09/HM39 for five digestion sets of both varieties.

Experiment identified the enzyme digestion efficiency in both varieties, and the results revealed that two varieties had a same enzyme digestion efficiency but different amplified bands. Five experimental sets and corresponding negative controls (without enzymes) were incubated at 37°C for 0.5 h, 1 h, 2 h, 4 h, 8 h for both varieties, respectively (Figure S1). The genomic DNA in negative controls was not digested while the samples in all experimental sets were thoroughly digested (Figure S1). After pre-amplification, selective amplification with 6 randomly selected primer-pairs and electrophoresis using polyacrylamide gels was subsequently conducted. The results revealed that digestion for 2 h had no difference with digestion for 4 and 8 h in amplified bands for both varieties, but that II-32B and Huhan-3 had many different amplified bands with the same primer-pair (Figure 1, Figure S2). Arrows 1, 3 and 4 represent bands only amplified in Huhan-3 and arrows 2 and 5 only represent bands that were amplified in II-32B (Figure 1). Selective amplification of the other 5 primer-pairs is shown in Figure S2.

### General DNA methylation levels of II-32B and Huhan-3 in different generations and treatments

A total of 3070 and 4739 MSAP loci were recorded in II-32B and Huhan-3 using the 256 primer-pairs, respectively (Table 1). In both varieties, unmethylated sites accounted for more than half of the CCGG sites (II-32B >56%, Huhan-3 >65%) (Table 3&4). The DNA methylation level of II-32B is higher than that of Huhan-3. Statistical results showed that II-32B has a significantly lower proportion of free methylated loci (Type I) (P<0.01) and a significantly higher proportion of both hemimethylated loci (Type III) (P<0.01) and hypermethylated loci (Type IV) (P<0.05) (Table 3&4, Table S2). Under normal cultivation and drought treatment, the general DNA methylation levels at G0 were about 39% in II-32B and about 31% in Huhan-3, respectively. At G6, the methylation level of II-32B declined to 31.78% under normal condition and recovered to 38.96% after drought treatment while Huhan-3 retained the G0 methylation level under both conditions. In addition, we found that II-32B had more differentially methylated loci (DML) between generations or / and between treatments (402, accounting for ~13.1% of total 3070 loci, Table 5) than Huhan-3 had (84, accounting for ~1.8% of total 4739 loci, Table 6). 

**Table 3:** DNA methylation patterns and levels of II-32B in different generations and treatments.

Generations	G0				G6			
Treatments	CK	Per (%)	DT	Per (%)	CK	Per (%)	DT	Per (%)
Type I (1,1)	1751	57.04	1738	56.61	1971	64.20	1750	57.00
Type II (1,0)	872	28.40	800	26.06	812	26.45	811	26.42
Type III (0,1)	232	7.56	245	7.98	247	8.05	248	8.08
Type IV (0,0)	215	7.00	287	9.35	40	1.30	261	8.50
Total bands	3070		3070		3070		3070	
Methylation level (%)	39.19		39.40		31.78		38.96	
Methylated sites (%)	42.96		43.39		35.80		43.00	
Free-methylated (%)	57.04		56.61		64.20		57.00	

**Table 4:** DNA methylation patterns and levels of Huhan-3 in different generations and treatments.

Generations	G0				G6			
Treatments	CK	Per (%)	DT	Per (%)	CK	Per (%)	DT	Per (%)
Type I (1,1)	3118	65.79	3122	65.88	3112	65.67	3145	66.36
Type II (1,0)	1317	27.79	1310	27.64	1317	27.79	1305	27.54
Type III (0,1)	274	5.78	263	5.55	272	5.74	264	5.57
Type IV (0,0)	30	0.63	44	0.93	38	0.80	25	0.53
Total bands	4739		4739		4739		4739	
Methylation level (%)	31.31		31.35		31.46		30.85	
Methylated sites (%)	34.21		34.12		34.33		33.64	
Free-methylated (%)	65.79		65.88		65.67		66.36	

**Table 5:** Directional and transgenerational DNA methylation changes in II-32B.

	CK vs. DT in G0	No change	Re-methylated	De-methylated
	Total loci	254	112	36
G0 *vs.* G6 in CK	No change	4	52	11
	Re-methylated	29	8	4
	De-methylated	221	52	21
G0 *vs.* G6 in DT	No change	219	83	28
	Re-methylated	13	0	8
	De-methylated	22	29	0

**Table 6:** Directional and transgenerational DNA methylation changes in Huhan-3.

	CK vs. DT in G0	No change	Re-methylated	De-methylated
	Total loci	33	30	21
G0 *vs.* G6 in CK	No change	25	6	5
	Re-methylated	6	24	0
	De-methylated	2	0	16
G0 *vs.* G6 in DT	No change	5	24	18
	Re-methylated	4	2	2
	De-methylated	24	4	1

### G6 had more variations in DNA methylation between CK and DT than G0

**Figure 2:**
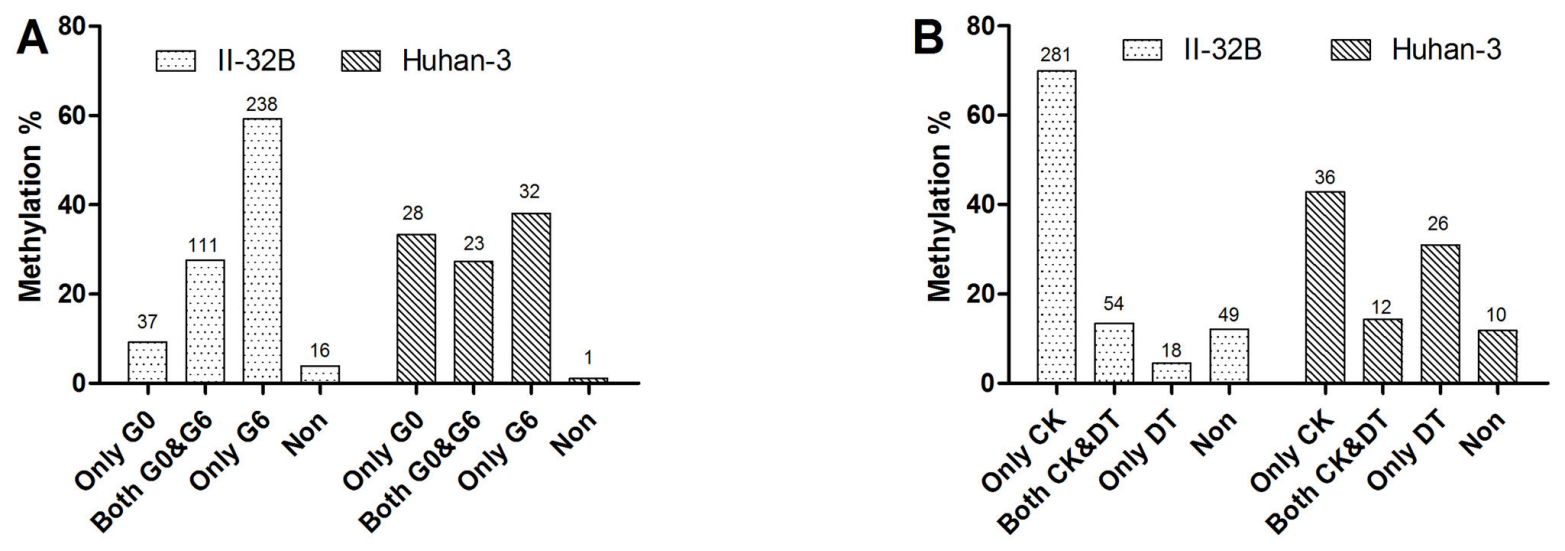
Further analysis of DNA methylation variations between generations and between treatments. (A) Comparison based on generations (G0 and G6). Only G0 means that DNA methylation variations occurred between CK and DT in G0, but not in G6; Both G0 and G6 means that DNA methylation variations occurred between CK and DT in both G0 and G6; Only G6 means that DNA methylation variations occurred between CK and DT in G6, but not at G0; Non means that no DNA methylation variations occurred between CK and DT in both G0 and G6, but G0 and G6 had different methylation pattern. (B) Comparison based on treatments (CK and DT). Only CK means that DNA methylation variations occurred between G0 and G6 under CK, but not under DT; Both CK and DT means that DNA methylation variations occurred between G0 and G6 under both CK and DT; Only DT means DNA methylation variations occurred between G0 and G6 under DT, but not under CK; Non means no DNA methylation variations occurred between G0 and G6 under both CK and DT, but CK and DT had different methylation pattern.

Further analysis of DNA methylation variations between generations (Figure 2A) and between treatments (Figure 2B) was performed to refine the methylation variation of both II-32B and Huhan-3 induced by drought stress. Based on the 402 and 84 DML in II-32B and Huhan-3, respectively, we observed that DNA methylation variations between CK and DT happened only in G6 more than only in G0 for both II-32B (238, 59.2%) and Huhan-3 (33, ~39.3%) (Figure 2A). 

### DNA methylation variations between G0 and G6 mainly happened in only CK set

**Figure 3:**
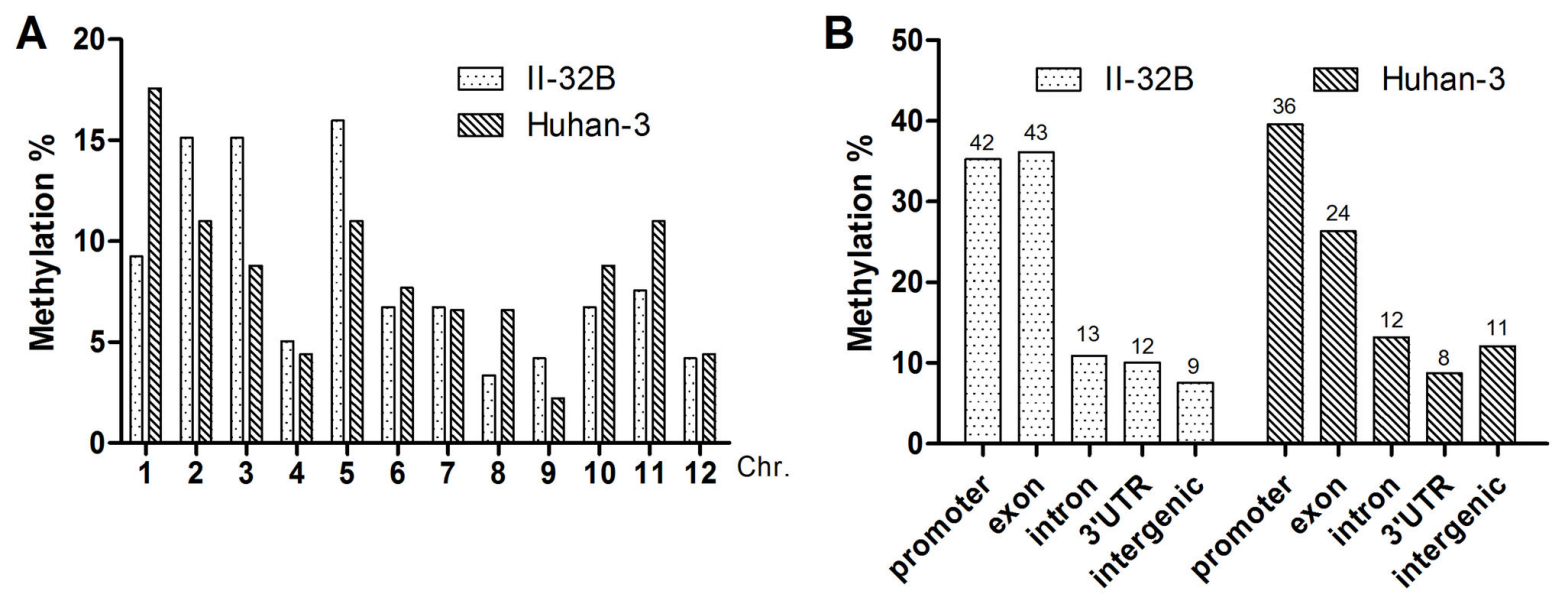
Distribution of the DML at chromosome level (A) and gene level (B)c.

Comparison based on treatments (CK and DT) in which G0 and G6 had variations in DNA methylation revealed that the DNA methylation variations between G0 and G6 mainly happened in only CK for both II-32B (302, 75.1%) and Huhan-3 (36, 42.9%) (Figure 3B). However, two generations of II-32B had larger differences in response to drought stress than Huhan-3 (Figure 3A). The DNA methylation status in both G0 and G6 of II-32B similarly changed when again subjected to drought stress, while G0 and G6 of Huhan-3 had less variation in CK, but had more variation in DT than that of II-32B (Figure 3B).

### Variations in DNA methylation directly induced by drought stress

Among the 402 DML in II-32B, 254 had no difference in DNA methylation status between CK and DT in G0 and these were considered unaffected by drought stress (Table 5). Changes between G0 and G6 or between CK and DT in G6 were caused by random factors, i.e. random methylation. The DNA methylation pattern was CK (G0) = DT (G0). Meanwhile, 112 and 36 DML became re-methylated (from type I to type II, III and IV; from type III to type II and IV) or de-methylated (from type II and IV to type I or type III; from type III to type I) after drought stress in G0, respectively. And most of these loci (83 in re-methylated status, accounting for 74.1%, and 28 in de-methylated status, accounting for 77.8%) still retained their re-methylated or de-methylated patterns after drought treatment in G6 with no change set in G0 vs. G6 in DT, which accounted for ~27.6% of total 402 DML (Table 5). Therefore, these 27.6% loci could be largely affected by drought stress, as they tend to be re-methylated or de-methylated in both G0 and G6 after drought stress. In sum, variations in DNA methylation were directly induced by drought stress. The DNA methylation pattern was CK (G0) ≠ DT (G0) = DT (G6).

Huhan-3 has only 84 DML, among which there were 33 loci affected by random factors. Meanwhile, 30 and 21 loci became re-methylated and de-methylated after drought stress in G0, respectively (Table 6). Compared to II-32B, there were 23 (79.3%) and 18 (~85.7%) that still re-methylated or de-methylated in G6 after drought treatment with no change set in G0 vs. G6 in DT and they accounted for ~48.8% of total 84 loci (Table 6). 48.8% of total 84 loci were directly induced by drought stress in Huhan-3.

### Transgenerational variations in DNA methylation response to drought stress

 Noticeably, in II-32B, 8 of 112 re-methylated loci already turned to re-methylated status in normal treatment in G6, with re-methylation set in G0 vs. G6 in CK (Table 5). Similarly, among the 36 de-methylated loci, 21 loci (~58.3%) kept their de-methylated status without the induction of drought stress of G6 (Table 5). All of the already turned sites accounted for ~7.2% (29) of total 402 loci. The re-methylated or de-methylated status kept in G6 without the induction of drought stress means their methylated status was determined through six generations’ entrainment under drought stress. More importantly, among the 8 and 21 loci, 4 and 9 loci had the DNA methylation pattern of CK (G0) ≠ DT (G0) = CK (G6) = DT (G6) (i.e. the DNA methylation status changed after drought stress in G0) and the changed status held in both CK and DT of G6. These belonged to loci whose variations in DNA methylation were directly induced by drought stress and the 13 loci accounted for ~3.2% of total 402 loci. This means that the variations in DNA methylation of these loci were directly induced by drought stress and might be stably inherited to progeny. 

In Huhan-3, there were 24 (80% of 30 re-methylated loci) and 16 (~76.2% of 21 de-methylated loci) loci that already turned into re-methylated or de-methylated status under CK in G6 (Table 6) and accounting for ~47.6% of total 84 loci, which indicated that a large proportion of these loci were of translational inheritance. Meanwhile, 15 of 24 loci and 10 of 16 loci had the DNA methylation pattern of CK (G0) ≠ DT (G0) = CK (G6) = DT (G6) which accounted for ~29.8% of total 84 loci. The variations in DNA methylation of these loci were directly induced by drought stress and could be stably inherited to progeny. 

### Drought induced genome-wide alteration in DNA methylation

To characterize the DML, 92 and 55 randomly selected fragments from 402 and 87 DML were sequenced for II-32B and Huhan-3, respectively. Then, the sequences of these DML were used as query searches against the nucleotide databases for homology on the Gramene web site to identify where DML happened. We defined the territory of a gene as the body (including its exons and introns) plus its putative promoter (the 3 Kb region upstream of the annotated transcription start site), while DNA between gene territories was designated as the intergenic region [5]. As shown in Figure 3A, the DML was widely distributed on all 12 chromosomes and chromosomes 4, 8, 9, and 12 had less DML than the other 8. Analysis on gene level revealed that DML of both varieties mainly distributed in the gene’s promoter and exon (especially in the first exon) region. (Figure 3B, Table S3).

### Genes with DML involved a wide range of functions

 A total of 110 and 80 genes corresponding to 92 and 55 DML in II-32B and Huhan-3 were determined according to their positions in the gene’s sequence, respectively. Some DML were located in the public region of two genes’ promoters and some had more than one highly matched site in the genome. All the genes were checked against the Rice Genome Annotation Project (Funded by NSF) and annotated using the rice genome annotation (version_7.0) (Table S3).

 In both varieties, based on the BLAST results, most of the genes were related to catalytic activity, hydrolase activity, transferase activity, etc. and which participate in protein modification, nucleoside metabolic and more (Table S4). Among these genes, 18 and 6 genes of II-32B and Huhan-3 were involved in the response to abiotic stimulus, biotic stimulus and endogenous stimulus in plants according to the GO analysis, respectively. One gene, LOC_Os03g44380, in which ectopic expression was reported to play an important role in ABA biosynthesis and drought response in Arabidopsis [51], has a different methylation status in its promoter between G0 and G6 under CK.

Among the 83 and 28 loci in II-32B that were directly induced by drought stress (Table 5), 16 and 8 of their loci were sequenced, respectively. 16 and 6 genes were found, including LOC_Os03g44380 and LOC_Os12g07810 in re-methylated loci, and LOC_Os03g57790 and LOC_Os05g49100 in de-methylated loci, which were involved in responding to stress according to the GO analysis (Table S5 & S6). In Huhan-3, 17 and 14 loci of 24 and 18 loci that were affected by drought stress were sequenced (Table 6) and 24 and 20 associate genes were found, respectively, including LOC_Os03g50210 in re-methylated loci and LOC_Os02g24190 in de-methylated loci which were involved in responding to stress (Table S7 & S8).

### Gene expression pattern changed after drought entrainment

**Figure 4:**
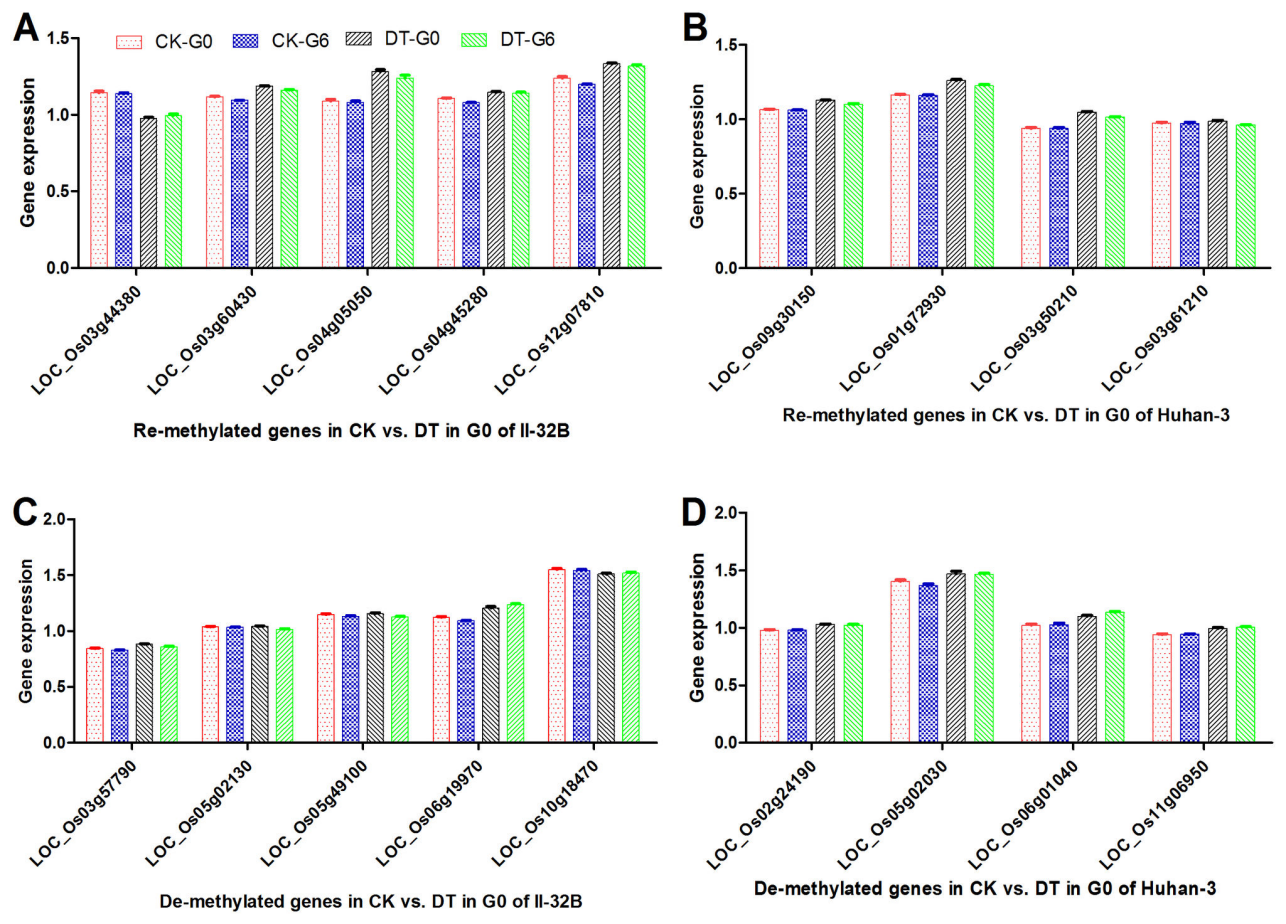
Expression of genes with DML. (A) Expression of genes which re-methylated in CK vs. DT in G0 of II-32B; (B) Expression of genes which re-methylated in CK vs. DT in G0 of Huhan-3; (C) Expression of genes which de-methylated in CK vs. DT in G0 of II-32B; (D) Expression of genes which de-methylated in CK vs. DT in G0 of Huhan-3.

To investigate the expression variation of the genes with DML, 18 genes’ expression were identified, including 5 re-methylated and de-methylated genes in CK vs. DT in G0 of II-32B, and 4 re-methylated and de-methylated genes in CK vs. DT in G0 of Huhan-3 (Figure 4, Table S5-S8). Analysis revealed that one re-methylated gene, LOC_Os03g44380, was down expressed in CK vs. DT in G0 of II-32B, while de-methylated genes, such as LOC_Os03g57790 and LOC_Os06g19970, were up expressed (Table 7). However, most re-methylated genes were up expressed, and some de-methylated genes were down expressed in II-32B. In Huhan-3, all the de-methylated genes were up expressed, but the re-methylated genes were also up expressed (Table 8).

**Table 7:** Expression of genes with DML in II-32B.

CK vs. DT in G0	G0 vs. G6		CK vs. DT	
	CK	DT	G0	G6
re-methylated genes				
LOC_Os03g44380			**↓	**↓
LOC_Os03g60430	**↓	**↓	**↑	**↑
LOC_Os04g05050			**↑	**↑
LOC_Os04g45280	**↓		**↑	**↑
LOC_Os12g07810	**↓		**↑	**↑
de-methylated genes				
LOC_Os03g57790	*↓	**↓	**↑	**↑
LOC_Os05g02130		**↓		*↓
LOC_Os05g49100		**↓		
LOC_Os06g19970	**↓		**↑	**↑
LOC_Os10g18470			**↓	

** P<0.01; * P<0.05; ↓ down-regulated; ↑ up-regulated.

**Table 8:** Expression of genes with DML in Huhan-3.

CK vs. DT in G0	G0 vs. G6		CK vs. DT	
	CK	DT	G0	G6
re-methylated genes				
LOC_Os09g30150		**↓	**↑	**↑
LOC_Os01g72930		**↓	**↑	**↑
LOC_Os03g50210		**↓	**↑	**↑
LOC_Os03g61210		**↓		
de-methylated genes				
LOC_Os02g24190			**↑	**↑
LOC_Os05g02030			*↑	**↑
LOC_Os06g01040		**↑	**↑	**↑
LOC_Os11g06950			**↑	**↑

** P<0.01; * P<0.05; ↓ down-regulated; ↑ up-regulated.

More interestingly, since they changed their expression after drought treatment in both generations of both varieties, most of the genes were in response to drought stress. However, many genes’ expression significantly differed between G0 and G6 in CK or (and) DT in II-32B (Table 7), such as LOC_Os03g60430 and LOC_Os03g57790, while all of the identified genes’ expression in Huhan-3 had no difference between G0 and G6 in CK, but had significant difference in DT (Table 8). G0 and G6 had a different gene expression pattern after being entrained for six generations and two varieties with different drought resistance levels had different variation patterns.

## Discussion

Because natural variation and subsequent phenotypic selection are the driving forces behind evolution, life scientists often impose constant selection forces on model organisms through simple environmental changes in laboratories to seek phenotypes of interest [52,53]. Biotic and abiotic environmental stresses such as diseases, drought, high salinity, heavy metals, nutrient deficiency and more could induce DNA methylation variation in rice [14,20,54-59]. Growing evidence has shown that epigenetic mechanisms play an essential role in orchestrating inheritable and reversible changes in gene expression in plants, without any changes in the primary DNA sequence [58,60-63]. To observe the relationship between drought stress and DNA methylation variation, our study subjected two common breeding varieties of rice with different drought sensitivities to constant drought stress for six generations. Our study differs from most previous studies, which experimented under benign greenhouse conditions or without select forces [53,64]. 

### Drought stress had significant cumulative effect on rice DNA methylome

Compared to G0, the DNA methylation patterns of both varieties changed after being cultivated successively for six generations under drought stress. The DNA methylation level and the proportion of methylated sites of G6 decreased largely in II-32B (Table 3). ~13.1% and ~1.8% of the total identified loci in II-32B and Huhan-3 were differentially methylated between generations or/and between treatments, respectively. A notable portion was directly induced by drought stress (27.6% in II-32B and 48.8% in Huhan-3, Table 5&6). The variations in DNA methylation included re-methylation and de-methylation events. This was consistent with previous studies, which found that environmental stresses induced DNA methylation changes in rice and other plants [13,14,20,54-59,65-67]. However, the result was compared to a study using peas that found water deficit increased DNA methylation level at CCGG sites by 40% [68]. Wang et al. reported that drought-induced, site-specific, genome-wide DNA methylation changes accounted for ~12.1% of the total methylated sites in the rice genome; DNA methylation change was measured at approximately the same proportion in II-32B, which is higher than Huhan-3 [14]. The difference of varieties might have led to the different results. In addition, the present study showed the DNA methylation variations between CK and DT happened in G6 more than in G0 (Figure 2A) and that the variations between G0 and G6 mostly occurred only in CK (Figure 2B). Results indicate that DNA methylation patterns that changed in G6 under CK, were preserved after entrainment under drought stress for six generations. When subjected to drought stress, G6 responded more flexibly than G0. This was an intriguing discovery and more rice varieties are needed to validate findings. 

### Direct drought stress-induced and transgenerational variations in DNA methylation

Some of the DNA methylation variations were directly induced via drought stress and a considerable proportion stably inherited their changed DNA methylation status in successive generation of G6. 27.6% of total 402 DML in II-32B and 48.8% of total 84 DML in Huhan-3, were directly induced by drought stress (Table 5&6). Furthermore, 3.2% and 29.8% of total DML in II-32B and Huhan-3, respectively, stably inherited their changed DNA methylation status to G6 with the DNA methylation pattern of CK (G0) ≠ DT (G0) = CK (G6) = DT (G6). Conclusions can also be inferred from previous studies, which stress that most directly induced DNA methylation changes are faithfully transmitted to offspring in asexual dandelions [13,27] and that nitrogen-deficiency and heavy metal stress could induce heritable alterations in DNA methylation [57,59]. These findings may be significant for understanding crop varietal improvement and plant evolution as epigenetic variations, which are induced by environmental stresses, could be inheritable and consequential to adaption and evolution in plants [13,25,59,69-72]. This transgenerational mechanism largely affected plant tolerance for rapidly changing environmental conditions and for the subject range of the ecological environment [11].

### Distinctive variations in DNA methylation between two varieties with different drought resistance levels

In the present study, we observed that in many respects the drought-resistant variety Huhan-3 displays a weaker response to drought stress than the drought-sensitive variety II-32B. First, II-32B had a higher general DNA methylation level (~39%) than Huhan-3 (~32%), because it had a lower proportion of free methylated loci (Type I) and a significantly higher proportion of hemimethylated loci (Type III) and hypermethylated loci (Type IV) (Table 3&4). This agreed with the study of horse gram that a higher hemimethylation status was found in the drought-sensitive genotype and that nonmethylation was higher in the drought-resistant genotype [73]. Second, II-32B had a higher proportion of DNA methylated sites (~43%) than Huhan-3 (~34%) (Table 3&4). Third, the DNA methylation pattern changed for 13.1% of the methylated sites in II-32B, compared to around 1.9% in Huhan-3. Fourth, 27.6% of DML was directly induced by drought stress in II-32B compared to 48.8% in Huhan-3 (Table 5&6). Lastly, 3.2% of DML were heritable in II-32B compared to 29.8% in Huhan-3. These results may indicate that the drought-resistant variety (Huhan-3) has a more stable methylome than the drought-sensitive variety (II-32B). Furthermore, drought entrainment had more effect on the drought-resistant variety than on the drought-sensitive variety. Further studies with more varieties are needed to validate these results. 

### Drought-induced genome-wide alteration in DNA methylation

Distribution of the DML revealed a genome-wide alteration in DNA methylation induced by drought stress. This is consistent with previous studies which found stress-induced widespread variations in DNA methylation in plants [14,15,74,75]. It is noteworthy that the DML was biased toward the promoter and exon (especially the first exon) of genes. We speculated that these genes were induced through environmental stresses, since DNA methylation in the promoter and gene body largely affect gene expression [5,10,76]. Functional analysis revealed that these genes were involved in a wide range of functions and participated in many important biological processes (Table S3&S4). More importantly, we found that 18 genes in II-32B and 6 genes in Huhan-3 responded to abiotic and biotic stress in plants. 

Additionally, other epigenetic marks, such as histone modifications and non-coding RNAs, also play important roles in responding to environmental stresses [23,77]. More researches should be performed to study their functions in responding to drought stress in our study.

### G0 and G6 had different gene expression patterns after entrainment

 Analysis revealed that some re-methylated genes’ expression like LOC_Os03g44380’s were down-regulated, while many de-methylated genes’ expression like LOC_Os03g57790’s were up-regulated (Table 7). At the same time, many re-methylated genes were up-regulated and some de-methylated genes were down-regulated. These findings contradict previous studies that found a negative correlation between DNA methylation and a gene’s expression [5,10,76]. Two reasons might have led to the different results: first, affecting a gene’s expression may need many clustered loci to be methylated or de-methylated, such as the CpG islands; second, other epigenetic mechanisms, such as histone modifications and non-coding RNAs, could affect genes’ expression.

G0 and G6 had different genes’ expression pattern in CK or (and) DT in both varieties suggesting that genes’ expression changed after drought entrainment in II-32B (Figure 4, Table 7 & 8). Meanwhile, Huhan-3 had different variation pattern with II-32B. When cultivated under normal conditions, there was no difference in genes’ expression between G0 and G6 of Huhan-3, while most of genes had different expression pattern between them when subjected to drought stress. However, dehydration avoidance, such as the effect of root system, and drought recovery which were another two important drought resistance strategies, were not investigated in our study, more experiment should be conducted to study them.

### Comparison of 20% PEG6000 treatment and natural drought stress

The present study used a 20% PEG6000 treatment to simulate drought stress in seedlings. Although lots of evidence indicated that PEG-simulated stress differed from natural drought stress, PEG could be used as a permeable mass to simulate water stress (osmotic stress) for studying dehydration tolerance, one of plants’ major drought resistance strategies. 

Under the same root osmotic potential with PEG treatment, the drought resistance of two plant varieties was only from dehydration tolerance. Thus, we could analyze DNA methylation variations between G0 and G6 in both varieties which had different drought resistance level, by univariate analysis. Meanwhile, seedlings cultivated in growth chamber could exclude the effects of exogenous environmental factors and show more consistent phenotype.

## Supporting Information

Figure S1 **Restriction digestion efficiency identification.** (A) II-32B; (B) Huhan-3. M represent EcoRⅠ/MspⅠ lane, H represent EcoRⅠ/HpaⅡ lane, CK was negative control.
(PDF)

Figure S2 **Selective amplification with primer-pairs of E15/HM37 (A), E08/HM310 (B), E07/HM37 (C), E06/HM312 (D) and E03/HM35 (E) for five digestion sets of both varieties.**
(PDF)

Table S1 **Primers used in qPCR.**
(XLSX)

Table S2 **Significance differences of band type proportions among the two varieties.**
(XLSX)

Table S3 **Genes related with loci that have variation(s) in DNA methylation between generations or/and between treatments in II-32B and Huhan-3.**
(XLSX)

Table S4 **GO analysis of genes related with DMP in II-32B and Huhan-3.**
(XLSX)

Table S5 **16 genes related to 16 of 83 loci which largely affected by drought stress in II-32B, re-methylated in CK vs. DT in G0.**
(XLSX)

Table S6 **6 genes related to 8 of 28 loci which largely affected by drought stress in II-32B, de-methylated in CK vs. DT in G0.**
(XLSX)

Table S7 **24 genes related to 17 of 24 loci which largely affected by drought stress in Huhan-3, re-methylated in CK vs. DT in G0.**
(XLSX)

Table S8 **20 genes related to14 of 18 loci which largely affected by drought stress in Huhan-3, de-methylated in CK vs. DT in G0.**
(XLSX)
