# Supplementary material for: Transgenerational Variations in DNA Methylation Induced by Drought Stress in Two Rice Varieties with Distinguished Difference to Drought Resistance
Source: PLoS One. 2013 Nov 11;8(11):e80253. doi: 10.1371/journal.pone.0080253 (PMC3823650; doi:10.1371/journal.pone.0080253)
Supplement: Figure S1 — Restriction digestion efficiency identification. (A) II-32B; (B) Huhan-3. M represent EcoRⅠ/MspⅠ lane, H represent EcoRⅠ/HpaⅡ lane, CK was negative control. (PDF) [file pone.0080253.s001.pdf]

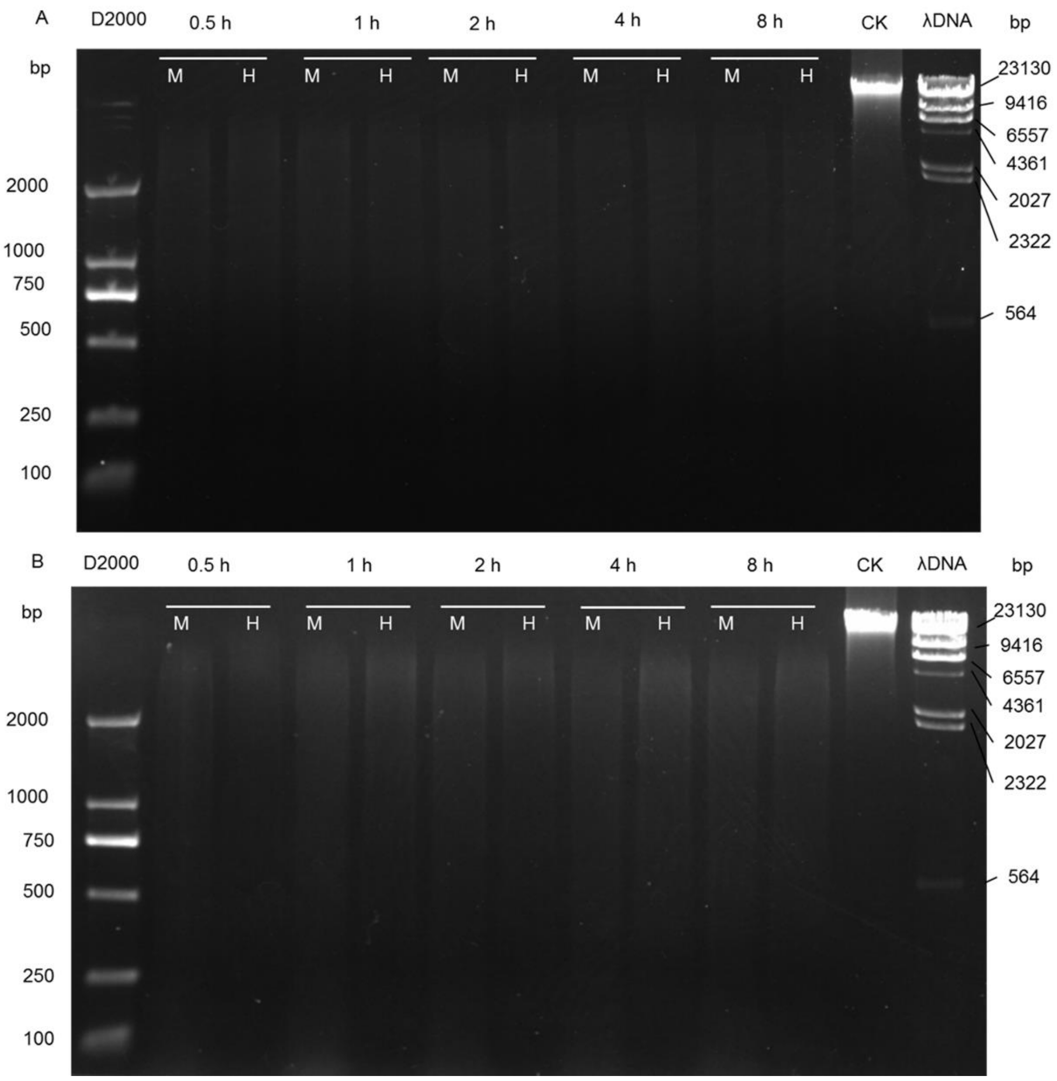

Fig S1 Restriction digestion efficiency identification. (A) II-32B; (B) Huhan-3.
M represent EcoRⅠ/MspⅠ lane, H represent EcoRⅠ/HpaⅡ lane, CK was negative control.
