# Supplementary material for: Transgenerational Variations in DNA Methylation Induced by Drought Stress in Two Rice Varieties with Distinguished Difference to Drought Resistance
Source: PLoS One. 2013 Nov 11;8(11):e80253. doi: 10.1371/journal.pone.0080253 (PMC3823650; doi:10.1371/journal.pone.0080253)
Supplement: Figure S2 — Selective amplification with primer-pairs of E15/HM37 (A), E08/HM310 (B), E07/HM37 (C), E06/HM312 (D) and E03/HM35 (E) for five digestion sets of both varieties. (PDF) [file pone.0080253.s002.pdf]

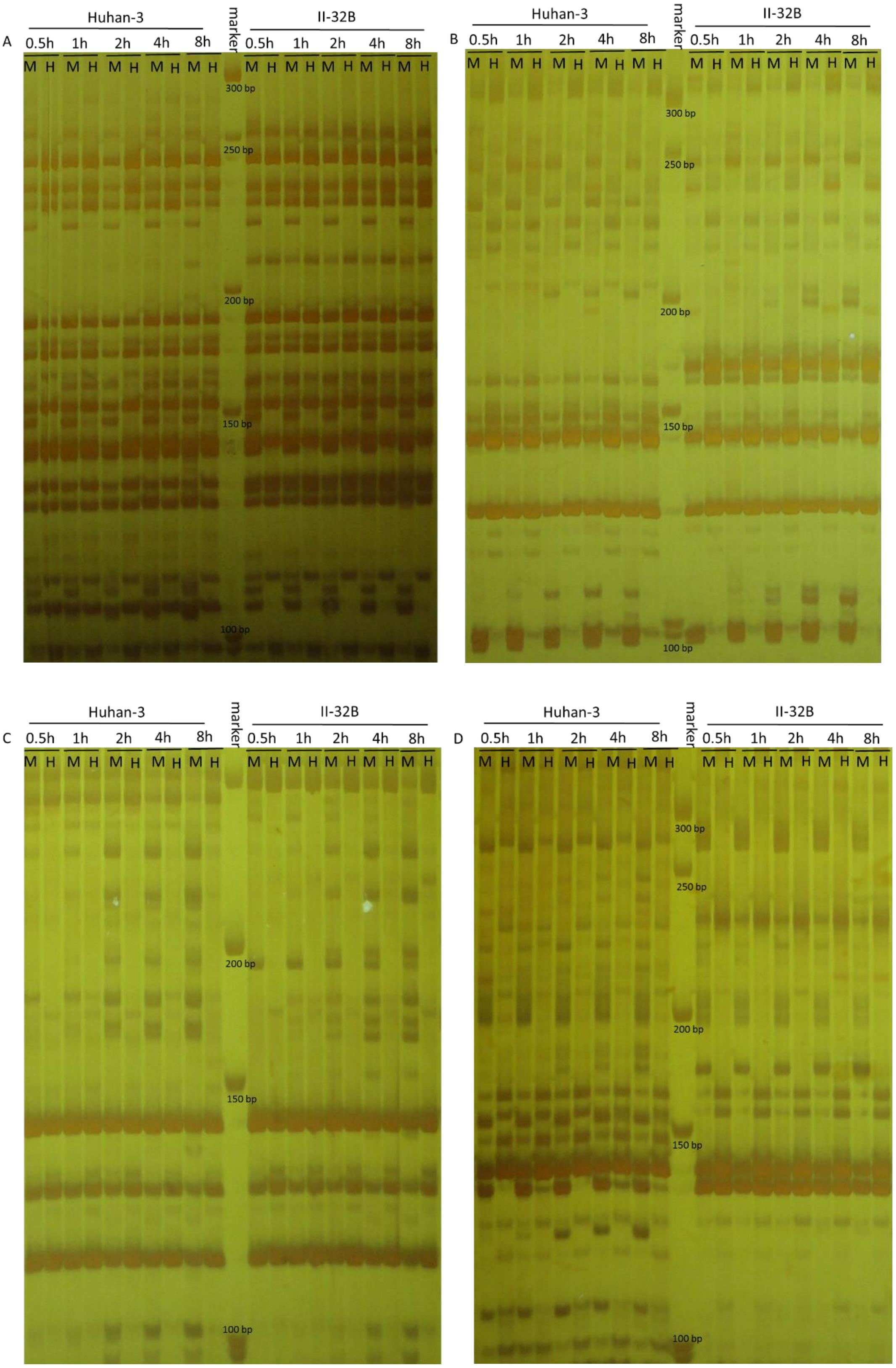

A
B
C
D
Huhan-3
II-32B
marker
0.5h
1h
2h
4h
8h
M
H
300 bp
250 bp
200 bp
150 bp
100 bp

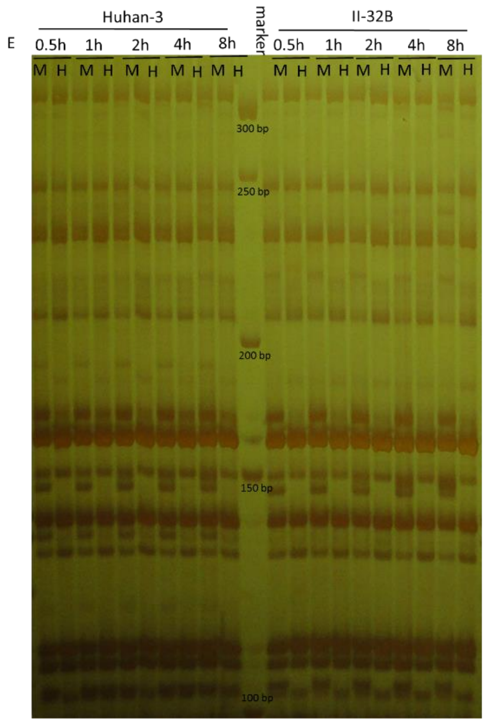

Fig S2 Selective amplification with primer-pairs of E15/HM37 (A), E08/HM310 (B), E07/HM37 (C), E06/HM312 (D) and E03/HM35 (E) for five digestion sets of both varieties.
